# Sentinel Lymph Node Evaluation: What the Radiologist Needs to Know

**DOI:** 10.3390/diagnostics9010012

**Published:** 2019-01-17

**Authors:** Gary J. Whitman, Raya H. AlHalawani, Niloofar Karbasian, Rajesh Krishnamurthy

**Affiliations:** 1 Department of Diagnostic Radiology, The University of Texas MD Anderson Cancer Center, Houston, TX 77030, USA; 2 Department of Radiology, The George Washington University Hospital, Washington, DC 20037, USA; ralhalawani@gwu.edu; 3 Department of Interventional Radiology, The University of Texas MD Anderson Cancer Center, Houston, TX 77030, USA; nkarbasian@mdanderson.org; 4 Department of Radiology, Nationwide Children’s Hospital, Columbus, OH 43205, USA; Rajesh.Krishnamurthy@nationwidechildrens.org

**Keywords:** breast cancer, axillary lymph node evaluation, sentinel lymph node biopsy

## Abstract

Axillary lymph node status is the single most important prognostic indicator in patients with breast cancer. Axillary lymph node dissection, the traditional method of staging breast cancer, is associated with significant morbidity. Sentinel lymph node biopsy has become standard in patients being treated for breast cancer with clinically negative lymph nodes. There is considerable variation in the medical literature regarding technical approaches to sentinel lymph node biopsy in patients with breast cancer. The purpose of this article is to describe our preferred approaches to sentinel lymph node biopsy with a review of the literature.

## 1. Introduction

**Figure 1:**
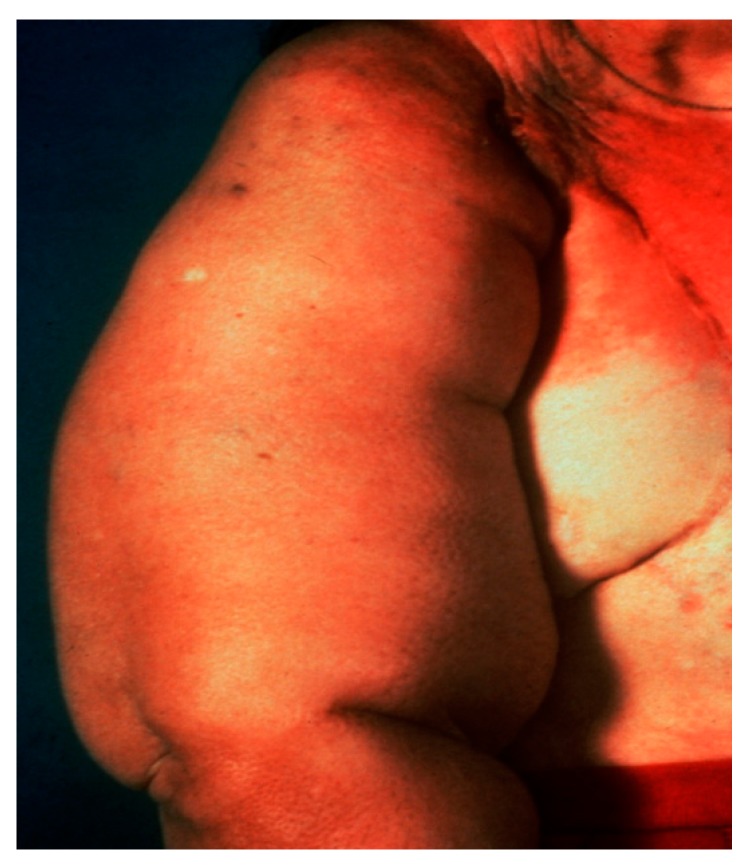
Lymphedema following axillary lymph node dissection.

Axillary lymph node status is the single most important prognostic indicator in patients with breast cancer [1]. Axillary lymph node dissection, the traditional method of staging breast cancer, is associated with significant morbidity, including injury to nerves and major blood vessels, infection, seroma formation and lymphedema (Figure 1) [2,3]. Diagnosis of early stage breast cancer with screening mammography has resulted in a significant decrease in the proportion of patients with positive axillary lymph nodes [3].

**Figure 2:**
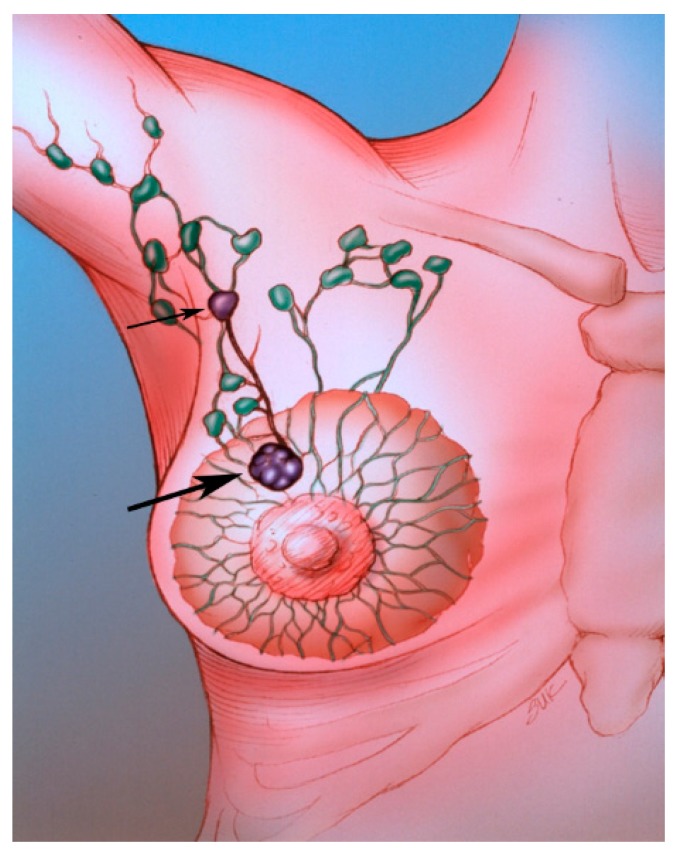
The SLN (small arrow) is the first lymph node draining the tumor (large arrow).

**Figure 3:**
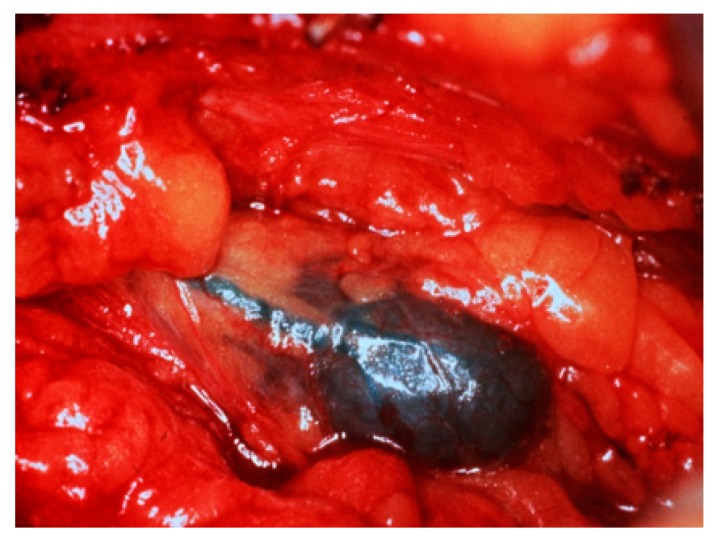
Intraoperative photograph following injection of isosulfan blue shows blue lymphatic channels leading to a blue SLN.

Sentinel lymph node biopsy (SLNB) has become standard in patients being treated for breast cancer with clinically negative lymph nodes [4]. The sentinel lymph node (SLN) concept (Figure 2), which states that the histologic status of the SLN is predictive of the status of the regional lymph nodes, is based on the orderly spread of tumor from the tumor bed to the regional lymph nodes. Sentinel lymph nodes (SLNs) are defined as the first group of lymph nodes draining the tumor bed. The SLNs can be located by injecting blue dye and/or radioactive material at the tumor site and subsequently, identifying a blue (Figure 3) and/or a radioactive lymph node in the axilla. Originally proposed in the management of penile cancer by Cabanas [5] in 1977, the SLN concept has been applied in patients with malignant melanoma [6] with considerable success. The American College of Surgeons Oncology Group (ACOSOG) Z011 trial compared two groups of clinical T1–2 N0 M0 breast cancer patients with a positive SLN [7]. The patients were treated with lumpectomy and opposing tangential field radiation therapy and adjuvant systemic therapy at the discretion of the treating physician. One group was randomized to SLNB without axillary dissection. The other group was randomized to SLNB followed by completion axillary dissection. The SLNB only group did not show inferior survival rates compared to the axillary lymph node dissection group [7].

Several studies have validated the SLN concept in patients with breast cancer [8,9,10,11,12]. In a multicenter validation study, the overall rate of identification of the SLN was 93% (in 413 of 443 patients). The accuracy of the sentinel node biopsy was 97% (392 of 405), the specificity was 100%, the sensitivity was 89% (101 of 114), the positive predictive value was 100%, the negative predictive value was 96% (291 of 304) and the sensitivity was 89% (101 of 114) [9,13].

A study by Goyal et al showed that the sensitivity of SLNB was 93.3% and the accuracy was 97.6% when using both blue dye and radioactive material. Some of the factors that were associated with failed localization included obesity, tumor location and non-visualization of the SLN on preoperative lymphoscintigraphy [14].

There have been conflicting data with regards to the use of indocyanine green fluorescence, contrast-enhanced ultrasound using microbubbles and MRI with superparamagnetic iron oxide nanoparticles in SLNB. Some have claimed that the techniques had limited ability to detect SLN metastases, while others had better results [15,16,17].

The purpose of this article is to describe our preferred approaches to SLNB with a review of the literature. In this paper, we discuss technical factors associated with SLNB and isotope characteristics, including size, volume and dose. We discuss injection techniques and examine the efficacy of SLNB in patients who have undergone prior excisional biopsies and prior percutaneous core needle biopsies. We review subareolar, subdermal and peritumoral injections as well as lymphoscintigraphy and we discuss the time between injection and lymphoscintigraphy or surgery. We review intraoperative SLNB, including gamma probe detection, pathology and pitfalls. We also discuss radiation safety issues.

## 2. Technical Factors

The various factors that influence the technical success of SLNB are listed in Table 1. There is considerable variation in the medical literature regarding technical approaches to SLNB in patients with breast cancer [13,15].

## 3. Isotope Characteristics

### 3.1. Size

A number of radiopharmaceuticals (Figure 3) including filtered and unfiltered Technetium (Tc)–99m-sulfur colloid, Tc-99m-colloidal albumin, Tc-99m-pertechnetate, Tc-99m-dextran and Tc-99m-antimony sulfide colloid have been used for injection. Sulfur colloid is favored in the United States. The ideal radioisotope is one that easily passes through the lymphatic channels leading to the SLN, allowing for visualization on early lymphoscintigraphic images. The ideal radioisotope should also be large enough to be trapped in the SLN and readily identifiable at subsequent surgical exploration. A comparison [16,17] of filtered (smaller colloid particle size, <0.22 µ) and unfiltered sulfur colloid (larger particle size, range 10–200 µ) is provided in Table 2. Studies have favored the use of unfiltered colloid over filtered colloid particles [18,19,20]. Linehan et al reported that unfiltered Tc-99m-sulfur colloid was superior to filtered Tc-99m-sulfur colloid [21].

In one study, 250 patients with operable breast tumors underwent lymphoscintigraphy before surgery. Three different size ranges of Tc-99m-labeled colloid particles (<50, <80 and 200–1000 nm) were used. Lymphoscintigraphy successfully revealed the lymphatic drainage in 245 of 250 patients (98%). The axillary SLN were identified in 240 patients (96%). SLNB correctly predicted the axillary node status in 234 of 240 patients (97.5%). Lymphoscintigraphy along with a gamma detection probe detected the SLN most easily and consistently when 200–1000 nm colloid particles were administered subdermally with an injection volume of 0.4 mL [22,21].

### 3.2. Volume

A large volume of injection (3–8 mL) has been associated with a better rate of SLN detection [21], probably related to acute expansion of the interstitial space. The use of unfiltered Tc-99m-sulfur colloid (larger particle size) with a larger injected volume allows for effective localization of the SLN [23,18].

### 3.3. Dose

The injected dose for SLNB in patients with breast cancer usually ranges from 0.3 to 3.0 mCi. At The University of Texas MD Anderson Cancer Center (UTMDACC), 0.5 mCi of Tc-99m-sulfur colloid is used if SLNB is performed on the same day as the surgical procedure. If the radiopharmaceutical is injected the day before surgery, the usual does is 2.5 mCi. One report has employed doses of up to 10 mCi of radiocolloid tracer [21,19].

## 4. Injection Techniques

For palpable lesions, direct peritumoral injection of the radiopharmaceutical is performed in the nuclear medicine suite. Four to six injections are performed with divided doses around the tumor. For nonpalpable lesions, mammographic and sonographic techniques for localization have been modified to accommodate the administration of the radiopharmaceutical. Imaging guidance allows for accurate localization of nonpalpable lesions as well as peritumoral injections of the radiopharmaceutical.

**Figure 4:**
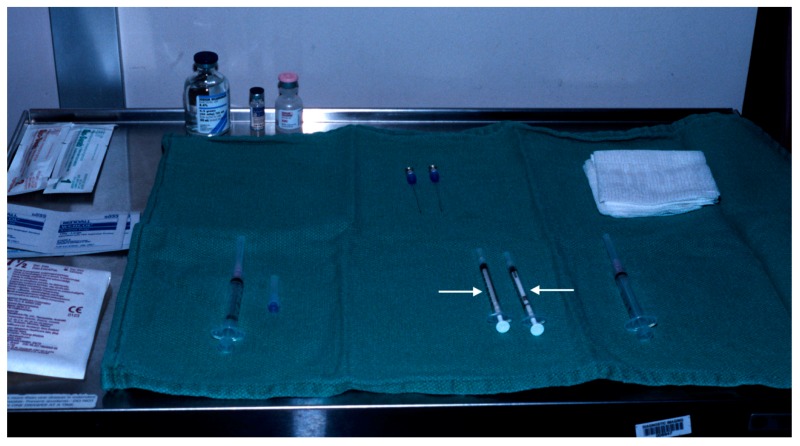
Procedure tray for mammographically-guided SLNB with two tuberculin syringes (arrows).

**Figure 5:**
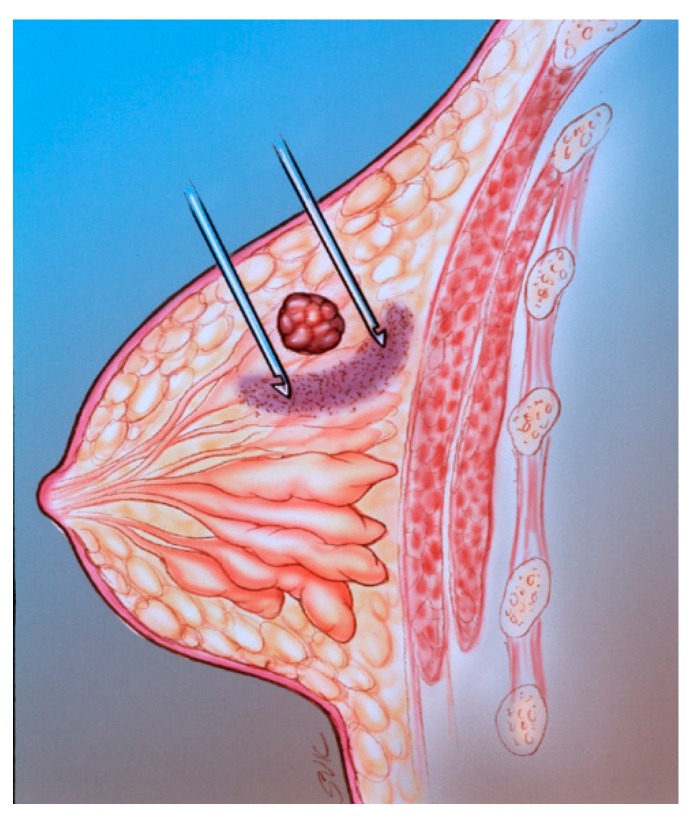
The diagram illustrates mammographically-guided SLNB. The pink semicircular region is the area of the peritumoral injection.

**Figure 6:**
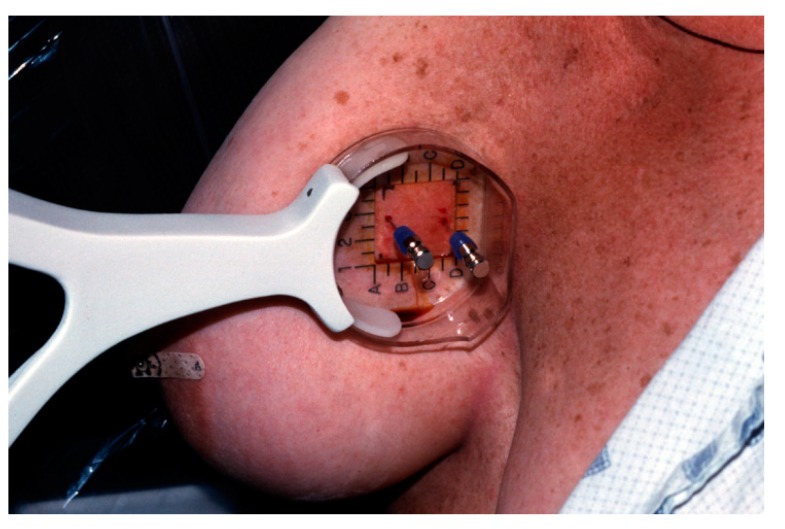
Mammographically-guided SLNB with two spinal needles.

**Figure 7:**
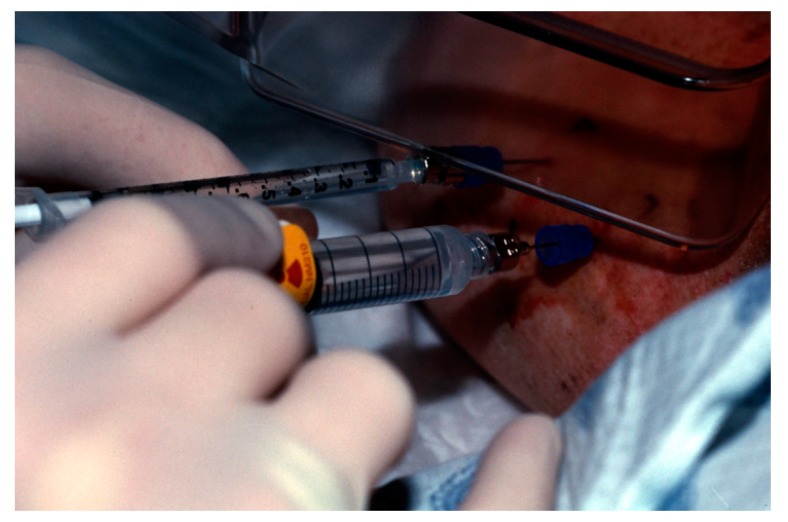
A tuberculin syringe containing saline is attached to the far needle. Tc-99m-sulfur colloid is injected into the near needle for SLNB.

Two tuberculin syringes are added to the standard procedure tray (Figure 4) for mammographically-guided SLNB. The radiopharmaceutical is kept in a lead container until the time of injection. Four mL of Tc-99m-sulfur colloid is injected in divided doses through the localizing needles (Figure 5). Hawkins, Homer, spinal (Figure 6) and Kopans needles have been used for mammographically–guided SLNB. After the injection of Tc-99m-sulfur colloid, saline is injected through tuberculin syringes to flush the localization needles (Figure 7).

**Figure 8:**
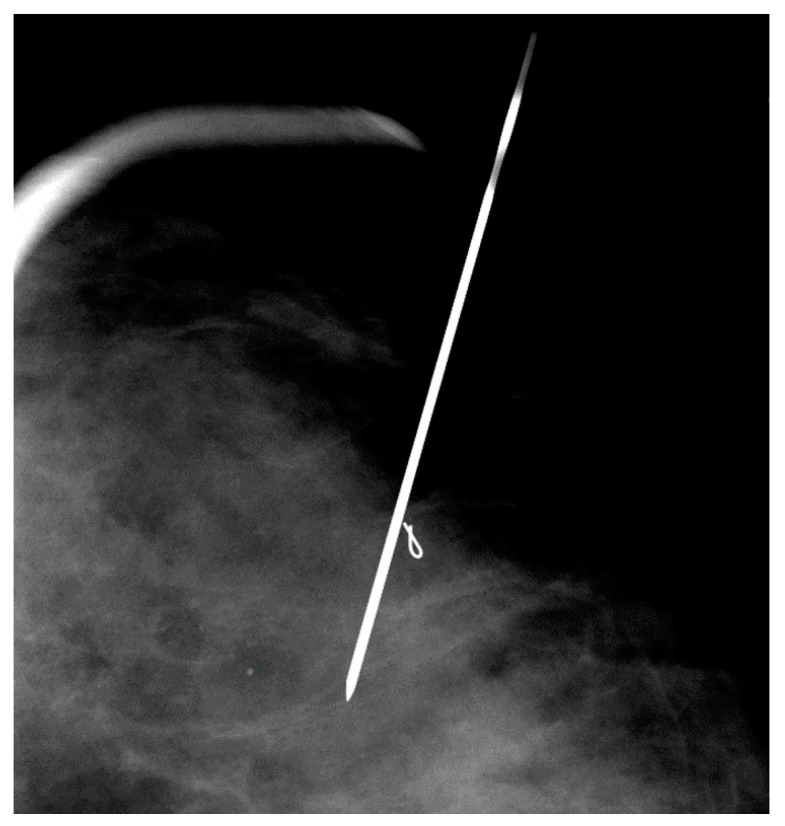
Mammographically-guided SLNB with one needle, targeting a clip marker.

**Figure 9:**
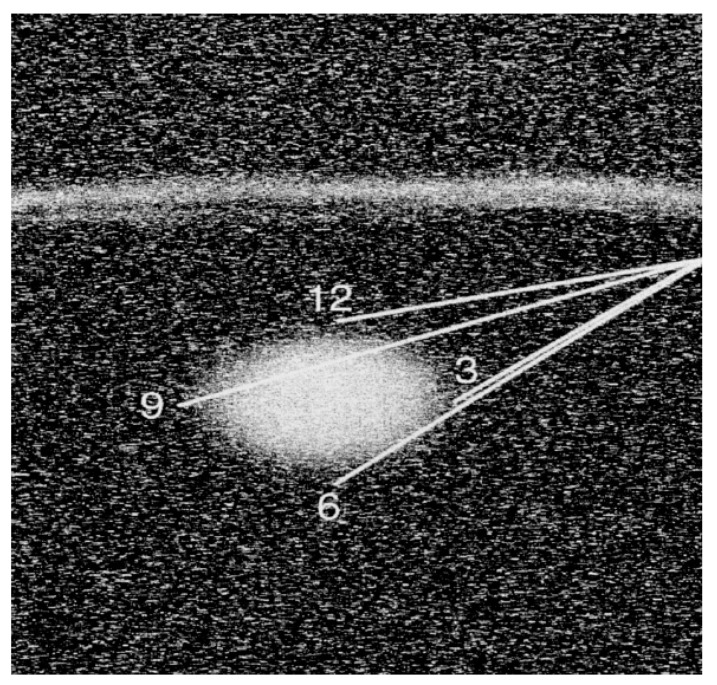
The diagram illustrates the technique for SLNB with sonographic guidance. Using one needle, peritumoral injections are performed at the three, six, nine and 12 o’clock positions.

**Figure 10:**
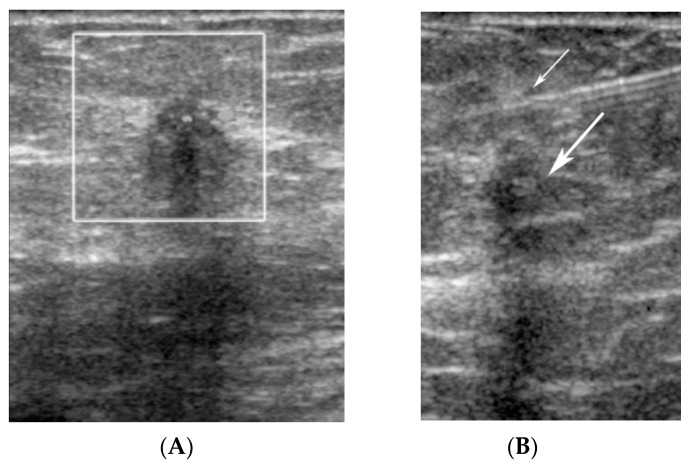
(**A**) A hypoechoic mass (white box), representing a known invasive ductal carcinoma, was targeted for SLNB with sonographic guidance. (**B**) The needle is seen at the 12 o’clock position (small arrow) relative to the malignant mass (large arrow).

Mammographically-guided SLNB has been performed on known cancers, suspicious masses and groups of calcifications, excisional biopsy cavities and metal clip markers (Figure 8) placed under stereotactic and sonographic guidance. While mammographically guided SLNB is often performed with two or more and needles, ultrasound-guided SLNB is usually performed with one needle (Figure 9 and Figure 10).

Currently, at UTMDACC, needle placement and injection for SLNB is performed with a combined approach with the needle placed in breast imaging under mammographic or sonographic guidance and the radiopharmaceutical injected by a nuclear medicine physician through or adjacent to the localization needle in the nuclear medicine suite. This approach requires the patient to be transported from breast imaging to nuclear medicine (one floor away) with a localizing needle in the breast. An advantage of the combined approach is that the radiopharmaceuticals can remain in the nuclear medicine area throughout the procedure.

## 5. The Efficacy of Sentinel Lymph Node Biopsy in Patients Who Have Undergone Prior Excisional Biopsy or Core Needle Biopsy

Iatrogenic disruption of normal lymphatic drainage pathways is an important consideration in assessing the accuracy of lymphatic mapping. The lymphatic drainage may be disrupted due to breast reduction surgery, implant placement, extensive injuries, burns, prior reconstructive surgery or surgery for hidradenitis. Conflicting reports have been made regarding the accuracy of SLNB in correctly predicting the status of axillary lymph nodes in patients with large excisional biopsies. Because of concerns over lymphatic disruption, several authors have suggested that prior excisional breast biopsy is a contraindication to SLNB [19]. Two studies have demonstrated that prior stereotactic core needle biopsy does not compromise the accuracy of SLNB, while prior surgical excisional biopsy has been associated with an increase in the number of studies in which a SLN was not identified [18]. Feldman et al suggested that excisional biopsy should be avoided prior to SLNB [19,20].

On the other hand, a study by Miner et al showed that the type of biopsy or the location of the primary lesion did not influence the ability to localize the SLN. In 57 patients who had axillary lymph node dissections, metastatic disease was identified in 23% (13 of 57). Axillary nodal status was accurately predicted in 98% (56 of 57) [20]. Successful SLN identification was shown to be independent of the biopsy technique (open surgical biopsy (95%) versus fine needle aspiration/core needle biopsy (96%)) [24].

In another study, the sensitivity of SLNB was high when using a higher dose of peritumoral radiocolloid tracer (10 mCi) combined with intradermal blue dye [21,25]. In patients who have undergone a prior excisional biopsy, it may be helpful to increase the dose and the number of radioisotope injections around the perimeter of the biopsy site.

## 6. Subareolar, Intradermal and Peritumoral Injections

**Figure 11:**
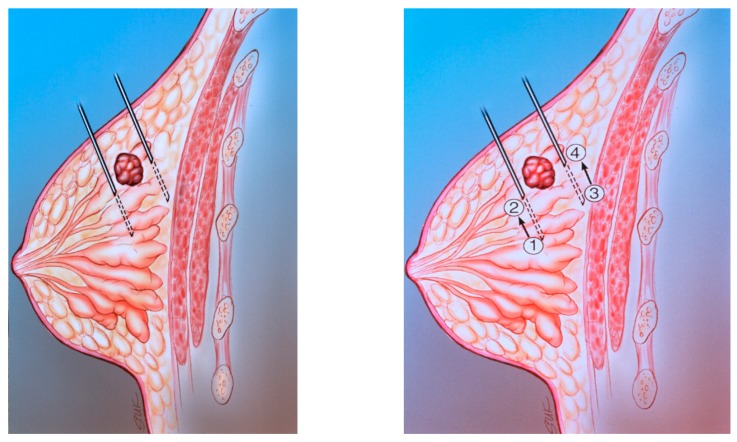
(**A**) The diagram shows peritumoral placement of two needles. (**B**) The localizing needles are placed with the tips at positions 1 and 3. The radiopharmaceutical is injected at positions 1 and 3. The needles are then withdrawn to positions 2 and 4. The radiopharmaceutical is then injected at positions 2 and 4.

Prior reports [26,27,28] have described a high degree of accuracy for intradermal injections of the isotope at the superolateral aspect of the tumor, probably secondary to communication between intraparenchymal and overlying dermal lymphatics. In a study comparing two different injection techniques (peritumoral and intradermal), lymphoscintigraphy was performed on 99 patients who underwent peritumoral and intradermal injections on separate days. Intradermal injections were performed either in the skin overlying the tumor or periareolar in the quadrant of the tumor. Ninety-four patients had positive peritumoral and/or intradermal accumulations. Fifty-two patients had complete concordance with axillary nodal uptake. In 30 patients, only peritumoral identification of the axillary nodes was successful and in nine patients, only intradermal identification of the axillary nodes was successful. Internal mammary nodes, visualized after peritumoral injection in nine patients, were not visualized by the intradermal technique. The authors concluded that intradermal injections are complementary to peritumoral injections (Figure 11) for patients with breast cancer [25].

**Figure 12:**
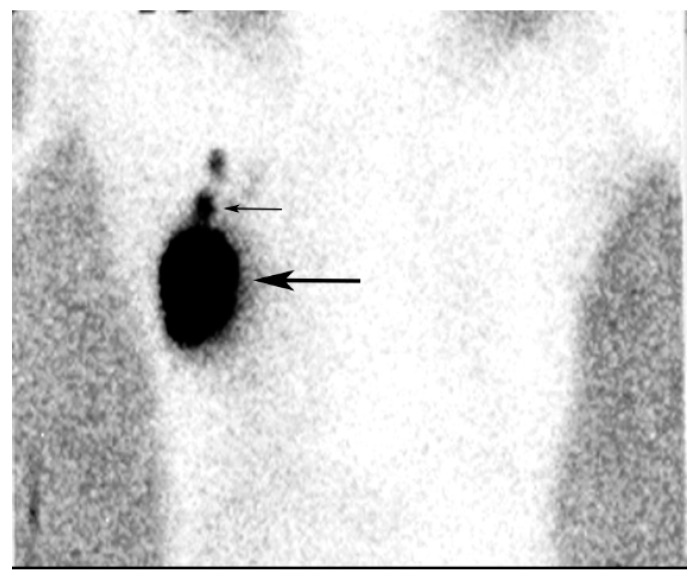
An injection site in the upper outer quadrant of the right breast (large arrow) is seen adjacent to a low axillary SLN (small arrow).

In another study, patients were injected with 1.0 mCi of Tc-99m-sulfur colloid (unfiltered) in the subareolar area of the tumor-bearing breast and an injection of 2 to 5cc of isosulfan blue was performed around the tumor. Thirty-two percent of the patients had history of previous excisional breast biopsies. Of the 69 lesions, 62 (89.9%) had SLNs located with the blue dye and 65 (94.2%) had SLNs located with the radiopharmaceutical. In four patients, the SLNs were not located with either method. All located SLNs were in the axilla. An average of 1.5 SLNs per patient were found in the 62 patients in which the SLNs were located with both methods and 23.2% of the SLNs had metastatic disease. All four patients in whom no SLNs were located with either method had undergone prior excisional biopsies. The authors concluded that subareolar injection of Tc-99m-sulfur colloid is as accurate as peritumoral injection of blue dye. Subareolar injection of the radioisotope avoids the problem of overlap of the injection site with the radioactive SLN (Figure 12), particularly in upper outer quadrant breast lesions [29].

## 7. Time between Injection and Lymphoscintigraphy or Surgery

The rate of lymphatic transit is variable, depending on the colloidal size, the injection volume and the site of injection. The lymphatic transit rate determines the time for imaging during lymphoscintigraphy and the time intervals between injection and surgical exploration. According to Dunnwald et al, the time to visualization of lymph nodes ranged from 1–120 min with a mean of 28 min [30]. Intradermal injections had a shorter transit time when compared to peritumoral injections.

## 8. Lymphoscintigraphy

**Figure 13:**
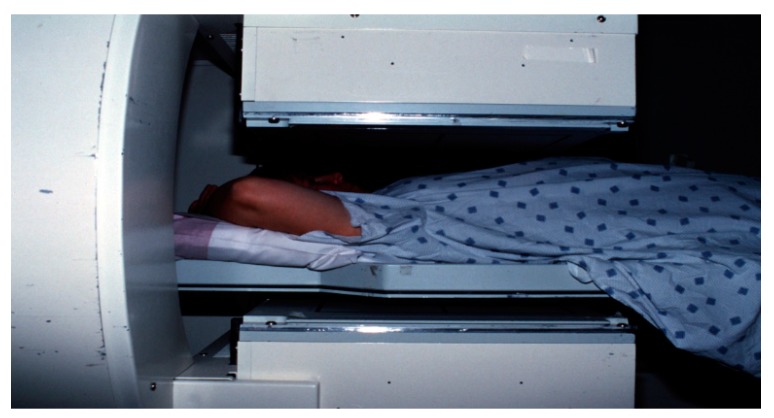
Anterior planar images following radiopharmaceutical injection.

**Figure 14:**
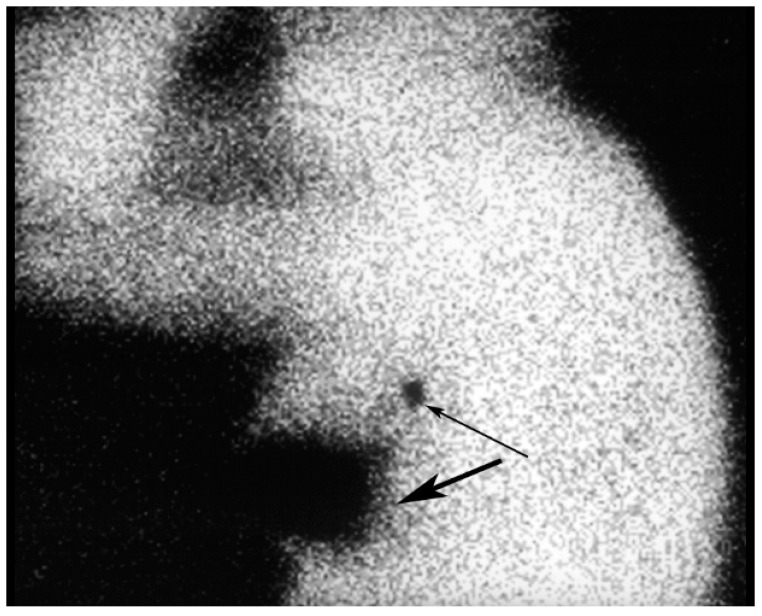
The lateral image shows the SLN (thin arrow). Marked uptake is noted in the tumor bed at the injection site (thick arrow).

Lymphoscintigraphy may be performed between 15 min to 24 h after administration of the radiopharmaceutical, depending on the composition of the radioisotope. The scanning protocol consists of dynamic and static images of the involved breast and the axilla in the anterior (Figure 13), anterior-oblique and lateral projections. Static planar images are usually obtained at 30 min and 3 h after radiopharmaceutical injection. The lymphatic channels are usually seen on the early scans, while the SLN (Figure 14) are usually best visualized on images obtained at 3h. At many centers, a skin marker is placed over the SLN. The skin marker may be useful in cases in which more than one lymph node takes up the radiopharmaceutical [31].

The rate of identification of the SLN by lymphoscintigraphy ranges from 75 to 98% [18,22,32,33]. Technical success with lymphoscintigraphy depends primarily on an adequate functional capacity of the sentinel node. Lymphoscintigraphy defines the pattern of lymph flow and facilitates the surgical approach to localization of the SLN. Lymphoscintigraphy can identify anomalous patterns of lymphatic drainage, which, in turn, may alter the surgical approach. In a multicenter validation study involving 443 patients, the SLN were outside of the axilla in 8% of cases and outside of level 1 nodes in 11% of cases. Three percent of positive sentinel nodes were in non-axillary locations [2]. After the SLN is visualized, transmission images using a cobalt (Co)-57 flood source are obtained to aid in anatomical localization [9].

In 70 unselected patients with breast cancer, lymphoscintigraphy detected internal mammary nodal update in 24 women. Internal mammary lymph node biopsy was attempted in all 24 patients (34%) and was successful in 15 patients. Five patients had metastatic involvement [21].

Some investigators have questioned the value of routine preoperative lymphoscintigraphy. Burak et al. stated that preoperative lymphoscintigraphy adds little additional information to intraoperative lymphatic mapping, thus making its routine not justified. There was no significant advantage with respect to SLN localization (91.7% versus 88.5%) in the group undergoing preoperative lymphoscintigraphy when compared with patients not undergoing preoperative lymphoscintigraphy [34]. Studies based on a meta-analysis of the available data suggests that a combination of preoperative lymphoscintigraphy with intraoperative dye-guided and gamma probe-guided methods achieve a higher rate of identification of SLN compared to any of the techniques alone [35]. Performing preoperative lymphoscintigraphy may be helpful for surgical planning in cases in which a SLN is not visualized. If a SLN is not seen on preoperative lymphoscintigraphy, the patient will usually be consented for axillary lymph node dissection.

Technical failures with radioisotope use have been reported in 5–30% of cases. Hence, a combined approach with a radioisotope and blue dye is recommended for SLNB. The combined approach has a reported technical success rate of 95% to 96% [36].

Failure of visualization of a SLN usually leads to axillary dissection. A false negative SLN occurs in 4.6% of cases [37]. The false negative results are due to either skip metastases or inaccurate localization of the sentinel node. According to Andersson et al, the risk of false negative was higher in patients with hormone receptor negative, multifocal tumors or those identified to have one sentinel lymph node [38]. Preoperative Tc-99m-colloidal albumin lymphoscintigraphy was performed in 130 consecutive patients with T1–T2, N0 breast cancer. Axillary focal accumulations were clearly identified on lymphoscintigraphy in 116 patients (89%). The failure rate was significantly higher in patients who had previous excisional biopsies (36%) than in those with palpable tumors in situ (4%) [18].

Summarizing the use lymphoscintigraphy in SLNB, consensus has yet to be reached for many topics such as tracer characteristics, injection volume and the site and the technique of administration. Lymphoscintigraphy by subdermal tracer administration is able to detect axillary lymph nodes in 98% of the cases but the method is accompanied by low visualization (2%) of drainage outside of the axilla, such as the internal mammary lymph nodes. While peritumoral administration is predominantly associated with late lymph node detection, the early appearance of SLN observed after subdermal and intratumoral tracer injections justifies obtaining early gamma camera images. The strategies for surgical identification of the SLN depend on the results of lymphoscintigraphy. Considering the first appearing node and the visualization of an afferent lymphatic vessel as the major criteria to identify the SLN, lymphoscintigraphy is thought to be conclusive in approximately 75% of the cases. When lymphoscintigraphy is not conclusive, injection with blue dye is recommended to aid in definitive identification of the SLN [39].

## 9. Intraoperative Sentinel Lymph Node Biopsy

**Figure 15:**
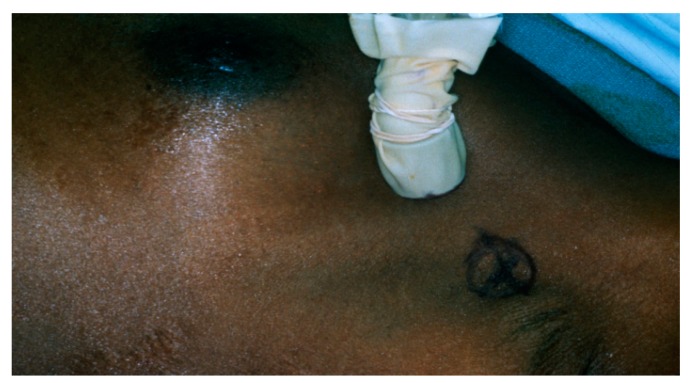
Intraoperative localization of a SLN using a Gamma Probe (Neoprobe 1500, Neoprobe Corp., Dublin, OH, USA). The mark on the skin of the axilla was made following lymphoscintigraphy.

The suitable timing for radiopharmaceutical injection ranges from 2 to 24 h prior to surgery [40]. Immediately prior to surgery, 2.5 to 7.5 mL of isosulfan blue dye is injected into the tumor bed in the operating room. A hand–held gamma detection probe is used to assist in SLN detection (Figure 15). Radioisotope injection allows the surgeon to localize the sentinel node prior to making an incision and blue dye enables the surgeon to visually identify the SLN or an associated lymphatic channel. Different criteria for the identification of SLN exist in the medical literature [13], including; lymph nodes with an acoustic signal, a discrete area of radiopharmaceutical uptake separate from the injection site with counts of at least 25 in 10 s, lymph nodes that have sentinel to non-sentinel lymph node count ratios of greater than 10; and greater than or equal to 25 counts over 10 s ex vivo in the resected specimen. The inability to find a “hot spot” or a blue lymph node is considered a technical failure.

Serial sectioning of the SLNB specimen is time-consuming and cumbersome. Careful serial sectioning has been enhanced by immunohistochemical staining with monoclonal antibody against low molecular weight cytokeratin with a high rate of detection of axillary lymph node micrometastases.

Recent studies have focused on intraoperative histologic evaluation of the SLN to give immediate feedback to the surgeon. One of these studies compared touch imprint cytology, frozen section analysis (FS) and rapid cytokeratin immunostain (RCI). Frozen section with RCI were found to have results closely approximating to the final pathological evaluation [41].

Another study compared real-time rt-PCR (reverse-transcription polymerase chain reaction) of mammoglobin and cytokeratin 19 to extensive histopathologic examination of axillary SLNs. This showed that the sensitivity of the (rt-PCR) assay was comparable to that of histopathologic examination of the entire SLN by serial sectioning at 1.5 to 2 mm [42].

## 10. Gamma Probe Detection

Tiourina et al. reviewed the necessary requirements for a gamma detection probe, such as: absolute sensitivity, spectral resolution, angular sensitivity and response ratio to the radioactive source. In addition, ergonomic characteristics are important [43].

Examples of practical performance simulations have been studied for five probes, showing that nodes at less than 115 mm from the injection site may be poorly localized, with even the best performing probe requiring at least 51 mm separation to allow for detection in the high background area near the injection site. The best probes allow for SLN localization between 20 to 30 mm closer to the injection site than the poorest performing probes [44].

## 11. Pathology

The resected lymph nodes are serially sectioned and submitted for routine pathologic evaluation. Standard staining is performed with hematoxylin and eosin. It has been demonstrated that meticulous analysis with serial sectioning of lymph nodes resulted in the identification of micrometastases [45] in 10–30% of lymph nodes that had been diagnosed as metastasis–free by routine histopathologic examination [46]. Immunohistochemical staining using the peroxidase–antiperoxidase technique with monoclonal antibody against low-molecular-weight cytokeratin has a high rate of detection of axillary lymph node micrometastases. Polymerase chain reaction [47] and new tumor markers have demonstrated greater sensitivity than immunohistochemistry for detection of micrometastases. In one study, 41 (10.6%) of the hematoxylin and eosin-negative patients were upstaged due to the detection of malignant cells by cytokeratin immunohistochemical staining of the SLNs. Evaluation of the SLNs with cytokeratin also shifted 10.6% of patients from stage I to stage II disease [48].

Serial sectioning is impractical for all axillary lymph nodes harvested from levels I and II but it is feasible if applied only to the SLNs. The percentage of patients found to have colonies of cells that were missed by routine sectioning corresponds closely to the percentage of “lymph node negative” patients who would be expected to relapse. The false negative rate for frozen section analysis of the SLN [49] is approximately 17%. The true clinical significance of axillary micrometastases will be determined by long-term follow-up [50,51,52].

## 12. Pitfalls

The technical failure rate of SLNB is about 5%. The best results have been with combined techniques (radiocolloid and blue dye). In patients in whom SLNB is deemed a technical failure, axillary dissections are performed. Some failures may be due to aberrant drainage patterns. In a meta-analysis of SLNB, the overall technical success rate of SLNB was 84% (762 lymph nodes), the accuracy was 98% (747/762) and the false negative rate was 5% (15/296). The highest technical success rates (*p* < 0.05) were reported with albumin radiocolloid or dye and radiocolloid (97 and 94%, respectively), with injection around an intact tumor (96%).

Factors associated with a technically difficult or failed SLNB procedures included inexperience of the surgeon, lateral hemisphere location, extensive axillary metastases and extranodal invasion [53]. The implications of primary drainage to aberrant lymph nodes are significant. Studies of lymph drainage patterns with lymphoscintigraphy suggest that the incidence of internal mammary metastases [54,55] without axillary metastases is 9%.

Diffusion of radioactivity injected around the tumor into the breast may preclude identification of a SLN near the tumor site due to radioactive shine-through. Shine–through occurs in lesions in the upper outer quadrant close to the axilla. SLNs located less than 115 mm from the injection site may be poorly localized secondary to high background activity. Another pitfall in lymphoscintigraphy is radiopharmaceutical within the needle hub simulating a lymph node.

Almost all investigators have reported an early learning curve in the practice of SLNB. A combination of radioisotope and blue dye techniques has resulted in a reduced technical failure rate [56]. Performance of SLNB results in an inability to locate a sentinel node in 38% of attempts during the learning phase [57].

## 13. Radiation Safety Issues

Low levels of radiation exposure [58] to patients and personnel are associated with SLNB. In one paper, tissue specimens obtained following SLNB were examined for residual radioactivity. Specimens with activity greater than the radiologic control level (RCL) of 0.002 microCi/g were considered radioactive. Twenty four (100%) of the specimens injected with radiopharmaceutical and 89 of 98 (91%) of the localized lymph nodes were found to be radioactive after surgery. The hands of the surgical team (*n* = 22 cases) were exposed to 9.4+/− 3.6 mrem/case [58] on measurement of the radiation exposure.

Each institution must establish safeguards for the use of radioactive materials. Each state’s radiation safety requirements must be satisfied. Preparation and injection of the radiopharmaceutical should take place under the auspices of approved nuclear medicine facilities. Routine badging of personnel handling the radioisotope and random radiation monitoring of the areas that may be exposed to radioactive contamination should be performed. Appropriate disposal of supplies and gloves that may be contaminated by radioactive isotopes is mandatory. Personal dosimetry is not required for members of the surgical team. In the operating room, additional shielding, monitoring devices or sufficient trash disposal protocols are not required.

## 14. Conclusions

SLNB using radioisotopes and/or blue dye provides accurate information regarding the axillary status in breast cancer, thereby avoiding the morbidity associated with axillary lymph node dissection. A combination of factors influence the technical success of the procedure, including the radioisotope injection, preoperative lymphoscintigraphy, performance of intraoperative lymph node mapping with blue dye, the characteristics of the intraoperative gamma detection probe, the methods of histopathological analysis and the experience of the clinical team. SLNB offers the opportunity for careful and meticulous analysis of a few selected lymph nodes, with decreased sampling error compared to standard histopathologic studies. These techniques may allow for the detection of micrometastases, thus improving the accuracy of staging in women with invasive breast cancer.

## Figures and Tables

**Table 1:** Technical Factors in Sentinel Lymph Node Biopsy.

**(1)** **Tumor localization techniques** Peritumoral injection with mammographic or sonographic guidance for nonpalpable lesions Direct injection around the needle and/or wire tip following mammographically-guided or ultrasound-guided needle localization Intradermal injection over the tumor site Direct injection around palpable masses **(2)** **Radiopharmaceutical properties** Choice of labeling agent: radioisotope and/or blue dye Size of the radiopharmaceutical: filtered versus unfiltered colloid Volume of radiopharmaceutical injected Interval between injection and surgery **(3)** **Experience of radiologists, surgeons and pathologists in SLNB** **(4)** **Criteria for identification of SLN at surgery with a gamma probe** **(5)** **Pathological methods for evaluating the SLN**

**Table 2:** Comparison of Filtered and Unfiltered Sulfur Colloid.

Type of Radiocolloid	Advantages	Disadvantages
Filtered (Smaller colloid particle, size <0.22 µ)	Increase visualization of afferent lymphatic channel coursing from primary tumor site Improved discrimination between SLNs and secondary lymph nodes on lymphoscintigraphy	Poor trapping, leading to a diffusely hot axillary bed
Unfiltered (larger colloid for particle, size 10–200 µ)	Excellent trapping properties in the SLN	Large colloidal particles may not migrate or migrate slowly from the tumor bed to the SLN
